# Evaluation of a web-based asthma self-management system: a randomised controlled pilot trial

**DOI:** 10.1186/s12890-015-0007-1

**Published:** 2015-02-25

**Authors:** John M Wiecha, William G Adams, Denis Rybin, Maria Rizzodepaoli, Jeremy Keller, Jayanti M Clay

**Affiliations:** Boston University School of Medicine, 72 East Concord St., B2900, Boston, MA 02118-2518 USA; Department of Pediatrics, Boston Medical Center, 1 BMC Place, Boston, MA 02118 USA; Boston University School of Public Health, 715 Albany St, Boston, MA 02118 USA; Department of Family Medicine, Boston Medical Center, 1 BMC Place, Boston, MA 02118 USA; Windsor Street Health Center/Cambridge Health Alliance, 119 Windsor Street, Cambridge, MA 02139 USA; Department of Obstetrics and Gynecology, Indiana University School of Medicine, 340 W 10th St #6200, Indianapolis, IN 46202 USA

## Abstract

**Background:**

Asthma is the most common chronic condition of childhood and disproportionately affects inner-city minority children. Low rates of asthma preventer medication adherence is a major contributor to poor asthma control in these patients. Web-based methods have potential to improve patient knowledge and medication adherence by providing interactive patient education, monitoring of symptoms and medication use, and by facilitation of communication and teamwork among patients and health care providers. Few studies have evaluated web-based asthma support environments using all of these potentially beneficial interventions. The multidimensional website created for this study, BostonBreathes, was designed to intervene on multiple levels, and was evaluated in a pilot trial.

**Methods:**

An interactive, engaging website for children with asthma was developed to promote adherence to asthma medications, provide a platform for teamwork between caregivers and patients, and to provide primary care providers with up-to-date symptom information and data on medication use. Fifty-eight (58) children primarily from inner city Boston with persistent-level asthma were randomised to either usual care or use of BostonBreathes. Subjects completed asthma education activities, and reported their symptoms and medication use. Primary care providers used a separate interface to monitor their patients’ website use, their reported symptoms and medication use, and were able to communicate online via a discussion board with their patients and with an asthma specialist.

**Results:**

After 6-months, reported wheezing improved significantly in both intervention and control groups, and there were significant improvements in the intervention group only in night-time awakening and parental loss of sleep, but there were no significant differences between intervention and control groups in these measures. Emergency room or acute visits to a physician for asthma did not significantly change in either group. Among the subgroup of subjects with low controller medication adherence at baseline, adherence improved significantly only in the intervention group. Knowledge of the purpose of controller medicine increased significantly in the intervention group, a statistically significant improvement over the control group.

**Conclusions:**

This pilot study suggests that a multidimensional web-based educational, monitoring, and communication platform may have positive influences on pediatric patients’ asthma-related knowledge and use of asthma preventer medications.

## Background

Pediatric asthma is a highly prevalent condition with significant risk for morbidity and mortality among children and adolescents [1,2] with particular impact on inner-city minority children [3-6]. In patients with persistent asthma symptoms, use of a controller (preventer) medication such as an inhaled corticosteroid improves symptoms and lung function, while reducing exacerbations and hospitalization [7]. Many children with asthma are frequently symptomatic, with clear indication that a higher step level of medication would be appropriate [3]. However, physician prescribing of, and patient adherence with, controller medications remains low [8], particularly among inner-city children from lower income families [9]. In addition, poor children with asthma are less likely to have access to asthma specialists [4].

Over the past two decades, strategies to improve adherence to controller medications have been tested using the Internet and other electronic modalities, with promising results [10-13]. Although newer technologies are being investigated [14], there continues to be interest in web-based approaches to promoting asthma self-care [15,16]. There is also evidence that cooperation, communication and coordination among health care providers can improve outcomes in chronic disease, [17-19] yet care often remains fragmented [20].

A recent systematic review of digital interventions for asthma care concluded that digital interventions show promise [13], although a recent meta-analysis also raised questions about the strength of evidence behind the effectiveness of telemedicine interventions for asthma [21]. Key intervention components to improve asthma outcomes identified included provision of asthma information and self-care education, asthma action plans, self-monitoring, immediate feedback from devices, messages and alerts to patients, games and quizzes, and availability for daily use [13]. However, none of these studies included all listed potentially effective elements in their intervention design.

This paper describes the design, implementation, feasibility and potential effectiveness of a digital intervention for children with asthma, BostonBreathes (BB), which included multiple strategies for improving asthma outcomes. In addition, reflecting the benefits of teamwork in asthma and other chronic illnesses, [19,22] BB leveraged the communication capabilities of the Internet to support clinical teamwork among health professionals involved in asthma care of the research subjects.

## Methods

### Design of website/pretesting/ theoretical basis

BostonBreathes (BB) is a web-based interactive asthma education, monitoring, and communication system designed to improve asthma care with 3 primary objectives:

1. improve adherence to asthma controller medications among children with asthma through education, self-monitoring, and rewards;
2. enhance teamwork between health care professionals caring for children with asthma by providing a communication platform; and
3. enhance primary care physician awareness of their asthma patients’ status in between clinical encounters.

The approach to behavior change was based in part on principles of social cognitive theory [23] and eHealth theoretical models [24]. The design process followed usability guidelines for development and testing of effective health-related websites [25].

**Figure 1:**
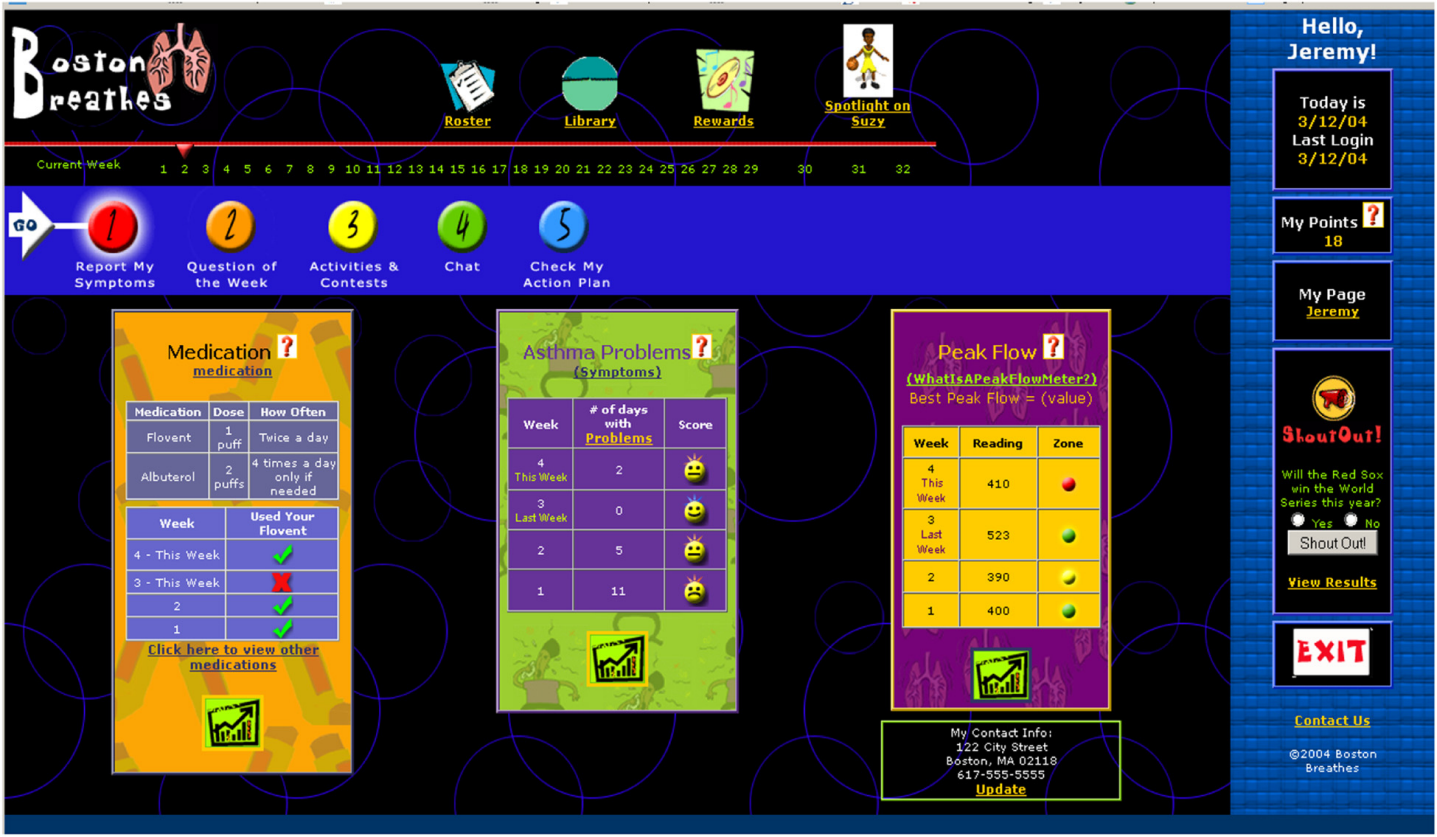
**BostonBreathes patient home page.**

After logging in with a password, subjects completed asthma education activities in a guided, deliberate sequence starting with reporting of asthma symptoms and impairment (see “Functions Supported by the BostonBreathes Websites” section). Completion of each function earned points, displayed on a counter on the webpage, which were redeemable for gift cards to a department store. Each data point entered was acknowledged by an interpretive response of the website: peak flow was coded according to action plan zone, and the website responded to symptoms data entered, or in response to a report of an appropriate level of use of controller medications, with facial expression tags (See Figure 1 for a screen capture of the patient’s web interface).

### Functions Supported by the BostonBreathes Websites

*Patient Website- Patient may complete the following each time they log on:*

- Enter measured peak flow and review web site response of corresponding zone (red, yellow, or green)
- Report symptoms (“problems”) (cough, wheeze, shortness of breath)
- Report limitations in activities, missed school, emergency room visits
- View and listen to asthma educational Flash animations
- Report medication use
- View graphs of data entered: symptoms, peak flow, medication use
- Complete asthma educational activities (puzzles, word searches, quizzes)
- Review asthma library of information
- View personal web pages of participants
- Edit personal webpage
- Participate in discussion board with peers
- Participate in discussion board with primary care provider
- Review points earned

*Primary Care Provider Website- provider can perform the following after login:*

- Review patient-level data on symptoms, medication usage, ER visits
- View patient graphical data of peak flow and symptoms
- Review peakflow data and peakflow zones
- Participate in private discussion board with patient and/or asthma nurse
- Submit queries to asthma nurse and/or asthma specialist physician
- Administrative Website
- Subject and physician registration and enrollment
- Monitoring of participation
- Alerts for dangerous values (red zone peak flow range)

A separate web interface for the primary care provider allowed access to summary forms of data (see “Functions Supported by the BostonBreathes Websites” section). An administrative interface supported enrollment and tracking functions. In recognition of the relationship between parental beliefs about asthma medications and medication adherence [26,27] parents were encouraged to participate with their child in the use of the website including educational activities.

### Study design

This was a prospective randomised pilot trial in which health care providers and a group of asthma patients were randomly assigned to one of three groups: Group 1 would receive usual care of their asthma from their primary care provider, Group 2 would use the full BB asthma monitoring and management website, including discussion boards to facilitate electronic teamwork; and Group 3 would use the website without the discussion boards. After randomisation, but before the study began, the two intervention groups were merged into Group 2 to enhance sample size and statistical power.

### Population and eligibility

The participating providers and patients were recruited from Boston community health centers, the Boston Medical Center, and other practices in the Boston area. Children with diagnoses of persistent asthma were identified by their primary care doctors, and were eligible if they were between the ages of 9 and 17. Caregivers were interviewed by phone and children were eligible if they could speak and read English, if they had a functioning Internet connection in the home, and if they had at least persistent-level severity of asthma or were on a controller-type medication.

Two baseline home visits (HV) were done on eligible patients. At HV1, baseline surveys were administered and a Doser- TM [28,29] device was attached to the controller medication metered dose inhaler to measure adherence rates, or the dose-counter number was recorded on controller-delivery devices using counters (such as fluticasone diskus delivery systems). After 2 weeks, the second HV was completed to pick up the Doser or record diskus numerical data, complete baseline surveys, randomise the patient, and train intervention subjects in the use of the BB website. All subsequent patients of each physician were placed in the same study group. Providers cared for patients in either the intervention or control group exclusively.

Children and parents were trained in the home by a research assistant who reviewed BB login procedures, established passwords, supervised data entry of current medications and doses into the website, and populated an asthma action plan visible on the website. The child was asked to navigate through the functions of the website to demonstrate competence, and then to make a posting to discussion boards.

Patients randomised into the control group received an asthma education manual [30], and peak flow meter, and otherwise received usual care from their physicians.

All patients in both groups received a PIKO (Ferraris Respiratory) digital peak flow meter with instructions on use, peak flow zones, and including a patient instructional guide. This device is an electronic hand held peak flow meter that displays peak expiratory flow rate. Patient peak flows were measured at HV1 and highest “personal best” value entered into the website which then automatically coded peak flow values subsequently entered by intervention group patients into red (<50% of best of FEV1, yellow (50-80% of FEV1), and green zones (>80% of FEV1) and displayed the zone on the website for immediate patient feedback. If values entered by patient consistently exceeded the initial value, it was adjusted upward to reflect the highest consistent peak flow number entered. Subjects were asked to use the PIKO in conjunction with website use. As a precaution, if a child entered a peak flow in their red zone, a pager carried by project staff at all times was activated automatically with an alert message. Upon enrollment, PCPs were requested to review and confirm the subject’s peak flow and symptom-based action plans.

Physicians with patients in the intervention group were trained in the use of BB website by one of the authors. To maintain provider awareness of patient use of the system, whenever their patient logged into BB, providers received an email notification with a hotlink to their login page on BB.

Every two months, all data entered by patients using the BB website system was reviewed by the project’s participating pediatric asthma specialist, and asthma nurse specialist. A summary of their conclusions and treatment recommendations, based on entered data, was posted to the private discussion board for review by the physician and patient and caregiver.

### Educational content

Educational content of the streaming videos included explanations of asthma and why it develops, how to mitigate impact on activities, use of controller and rescue medications, triggers, smoking, pets, action plans, and peak flow meters. Videos can be viewed at: http://www.bu.edu/fammed/bostonbreathes/menu.htm.

### Symptom assessment

Asthma symptoms, and behavioral impacts, over the preceding 2 weeks were measured at baseline and at 6 month time points using a validated questionnaire [31]. Adherence to controller medications was measured with a DOSER [29] which records number of actuations per day for 30 days and has been validated in comparison to self-report and canister weight testing [28].

Subjects received the DOSER at baseline and used it on their metered dose inhaler for 25 days. For those subjects using the Advair discus, the built in counting mechanism was recorded. For subjects using oral medications, pill counts were performed. Knowledge of purpose of medications was determined by asking a subgroup of subjects (all intervention and control subjects enrolled after a specific date), in an open-ended question format, to state the purpose of their controller medications. Results were recorded, blinded and post-coded as to accurate identification of the purpose of the medication. Patient and provider utilization of the websites was measured during the study period. An 8 item instrument was developed to measure confidence with a computer and the Internet. The instrument requested self-reported ability to perform basic functions in three domains key to BostonBreathes: computer operations, word processing, and Internet. The responses were scored using a 5-point Likert scale.

### Technical specifications

The website was built in ASP.Net 1.1 with a Microsoft Sql Server Database. Encryption was via a 256B encryption algorithm, and passwords were encrypted with a 1 way hash so they could not be decrypted. The system was designed to enable future population of an electronic medical record via the HL-7 data standard.

### Statistical methods

Because continuous variables of interest were not always normally distributed we performed both non-parametric and parametric analyses. The results of these analyses were very similar and led to the same conclusions. In order to simplify presentation of the results we only reported parametric analyses.

The baseline demographics were compared using two-sample *T*-test for all continuous variables and Fisher’s Exact test for categorical variables.

To assess the change over time in continuous variables within each intervention group, which were non-normally distributed, we used the Signed Rank test; and to compare the change across the intervention groups we used the Wilcoxon test. We used McNemar’s test to assess difference in marginal proportions over time for dichotomous variables within each group. Then, we compared the proportion of subjects who improved from baseline to month 6 across intervention groups using Fisher’s Exact.

Due to attrition we tested whether loss to follow-up was random (ie, deviation from the assumption of “missingness completely at random”) using the permutation test [32]. All statistical analyses were performed using SAS software, version 9.2 (SAS Institute Inc, Cary, NC, USA). P-values below 0.05 were considered to be significant. Written informed consent for participation in the study was obtained from all participants and their parent or guardian, and the study was reviewed for human subject protection and approved by the Boston University Medical Campus Institutional Review Board.

## Results

**Figure 2:**
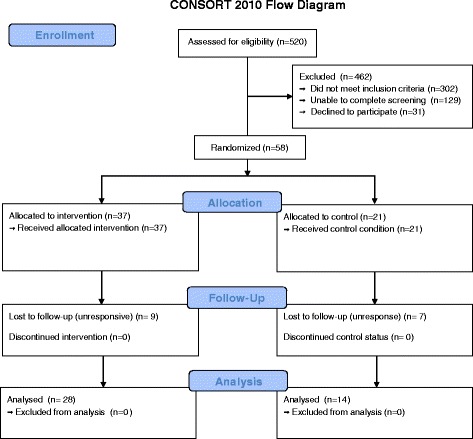
**Consort Diagram.**

983 children with asthma were identified through automated medical record review. Of these, 520 (52.9%) were able to be reached and had an eligible physician, 391 (75.2%) completed a phone screening, and 89 (22.8%) were deemed eligible for the study. Of the 89 eligible subjects, 31 declined to participate leaving 58 (65%) enrolled with 21 randomized to the control group, and 37 randomized to the intervention group. See Figure 2 for Consort diagram.

At the 6 month end-point, the control group retained 14 (66.7%) of enrolled subjects, and intervention group retained 28 (75.7%) of subjects. The other subjects were lost to follow-up before the 6 month end point. Analysis of the randomness of missing data for days of wheeze, days had to slow down, nights woke-up, days limited activity, days parent lose sleep and days missed school for asthma using the permutation test indicated no significant dependence of drop out on the outcome values. Thirteen [13] physicians and 1 NP participated in the study of whom 61% were female, the mean age was 45 years old, 95% were located in an urban practice area, 22% were family physicians and 78% were pediatricians or pediatric NP.

Characteristics of the subjects are described in Table 1. Overall, 58.6% of subjects were African-American, and 32.6% of household reported earning less than $15,000 per year. Most subjects (87.5%) had some form of health insurance. Parents of control subjects were slightly more likely to report high school education or above (78.4% of intervention subject parents, versus 100% of control parents, P = 0.041).

**Table 1:** **Sample Characteristics**

**Characteristic**	**Intervention, (N = 37)**	**Control, (N = 21)**	**P**
**Demographics**			
Age (years)			
Mean	11.9 ± 2.0	12.9 ± 3.0	0.13
Median and Range	12 (8–16)	14 (7–17)	
Male gender	22 (59.5%)	12 (57.1%)	1.0
Race			
Hispanic	4 (10.8%)	2 (9.5%)	0.70
Black	20 (54.1%)	14 (66.7%)	
White	8 (21.6%)	2 (9.5%)	
Other	5 (13.5%)	3 (14.3%)	
Parental Education: at least High School completion	29 (78.4%)	20 (100%)	0.041
Employed caregiver in household	29 (78.4%)	13 (61.9%)	0.23
Total family Income under $15,000	9 (28.1%)	6 (42.9%)	0.50
Child covered by health insurance	32 (91.4%)	17 (81%)	0.41
**Home Environment**			
Cockroaches at home	8 (22.9%)	7 (33.3%)	0.53
Dog at home	6 (17.1%)	3 (14.3%)	1.0
Cat at home	6 (17.1%)	9 (42.9%)	0.06
Pet rodent at home	3 (8.6%)	2 (9.5%)	1.0
Smokers at home	8 (22.9%)	9 (42.9%)	0.14
**Computer Use**			
Computer Competence Score, self reported (1–5), Mean (SD)	4.3 ± 0.6	4.6 ± 0.5	0.07
Hours/day on computer, Mean (SD)	2.0 + 1.8	1.6 + 1.6	0.51
Type of Internet Connection			
Dial-Up	10 (32.3%)	2 (13.3%)	0.29
Broadband	21 (67.7%)	13 (86.7%)	
Able to log onto Boston Breathes website			
With assistance	2 (7.1%)	2 (11.1%)	0.64
Without assistance	26 (92.9%)	16 (88.9%)	

**Figure 3:**
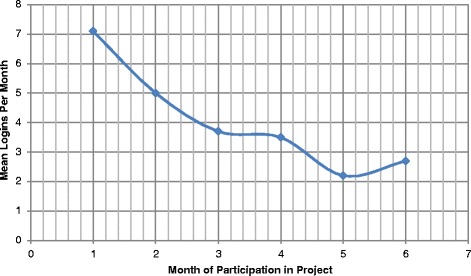
**Subject Logins to BostonBreathes Website, by Month.**

Intervention subjects accessed the BostonBreathes site on average 7 times per month in the first month of the study, with decrease over time (Figure 3).

### Clinical outcomes

Reported days of wheezing improved in both groups at the 6- month endpoint (−1.4 days of wheezing per 2 weeks in intervention subjects, and −4.2 days in control subjects), and patient awakening and parental loss of sleep improved significantly only in the intervention group (−0.8 and −0.6 days change per 2 weeks respectively at 6 months). However, there were no significant differences between groups in the changes of these measures (Table 2). Emergency room or acute visits to a physician for asthma also did not significantly change in either group over the study period.

**Table 2:** **Changes in Clinical Outcomes, Intervention vs. Control Group**

	**Intervention, N = 28**			**Control, N = 14**			
**Symptoms over previous 2 weeks**	**Mean at baseline**	**Mean change at 6 months**	**P-value**	**Mean at baseline**	**Mean change at 6 month**	**P-value**	**P-value for differences in changes**
Days of wheeze	2.9	−1.4	0.03	5.1	−4.2	0.004	0.10
Days had to slow down	2.5	−1.4	0.07	1.1	−0.4	0.48	0.79
Nights woke-up	1.2	−0.8	0.04	1.9	−1.0	0.06	0.66
Days limited activity from asthma	0.6	−0.2	0.99	2.0	−1.8	0.13	0.14
Days parent lose sleep	0.9	−0.6	0.01	0.9	−0.6	0.13	0.71
Days missed school for asthma	0.2	−0.2	0.38	0.4	−0.4	0.25	0.31
	Baseline N(%)	6-Month N (%)	p-value	Baseline N(%)	6-Month N (%)	P-value	P-value for differences in changes
Acute asthma-related PCP or ER visit in prior 2 months	5 (19.2)	1 (3.9)	0.18	1 (7.1)	1 (7.1)	0.99	0.64
**Primary controller compliance (%)**	Mean at baseline	Mean Change at 6 months	P-value	Mean at baseline	Mean Change at 6 month	P-value	P-value for differences in changes
All subjects (I = 21, C = 9)	38.0	+11.2	0.30	45.9	−4.4	0.81	0.46
High risk subjects* (I = 15, C = 8)	16.3	+29.8	0.01	39.2	−5.0	0.81	0.10
**Asthma Knowledge**	Baseline N(%)	6-Month N (%)	P-value	Baseline N(%)	6-Month N (%)	P-value	P-value for differences in changes
Correctly Described Purpose of Personal Controller Medicine (I = 12, C = 8)	4 (33.3)	9 (75.0)	0.03	4 (50.0)	3 (37.5)	0.25	0.03

*Those subjects with a baseline controller adherence of <75%.

Among the subgroup of subjects with low (<75%) controller medication adherence at baseline, controller medication adherence at 6 months improved significantly only in the intervention group.

Knowledge of the purpose of controller medicine increased significantly in the intervention group subset, but not in the control subset, a statistically significant difference favoring the intervention group.

### Communication, teamwork, and messaging outcomes

The most frequent messaging activity was between patients and the asthma nurse specialist, with less activity by the PCP (Table 3). The most frequent message content was encouragement of medication adherence and website use (Table 4), followed by discussion of asthma educational content, discussion of asthma symptoms, or issues regarding peak flow readings or measurement. Socializing on non-medical topics was very common. Explicit coordination of care represented about 5% of posting.

**Table 3:** **Summary by Sender of Private Chat Messages**

**Originator of message**	**Total # of messages sent, N (%)**
Asthma nurse	235 (38.7)
Asthma specialist	4 (7.0)
PCP	100 (16.5)
Patient	216 (35.6)
BB team	36 (5.9)
Parent	12 (2.0)
Blank/None	4 (7.0)

**Table 4:** **Summary by Content of Message**

**Primary Message Content**	**Total # of postings, N (%)**
Medication adherence encouraged or inquired by MD or RN	82 (13.5)
Encouraged patient to use BostonBreathes	78 (12.9)
Asthma education for patient	74 (12.2)
Asthma symptom or Peak Flow- addressed or asked by MD/RN	68 (11.2)
Socializing	61 (10.1)
Subject telling progress- asthma mentioned	42 (6.9)
Doctor/nurse providing general encouragement- positive feedback	38 (6.3)
Team review summary	29 (4.8)
Medication adherence issue addressed by patient	28 (4.6)
Coordination of care	21 (3.5)
Asthma symptoms or peak flow reported by patient	20 (3.3)
Non asthma illness posting	11 (1.8)
Socializing (doctor posting)	11 (1.8)
Appointment reminder for patient or patient asked to make appointment	10 (1.7)
Acknowledgement of a team posting by PCP or patient	7 (1.2)
Discussion of incentive points	7 (1.2)
Issue with Peak Flow meter	4 (0.7)
Blank message	4 (0.7)
Medicine dose change or medicine change discussed	2 (0.3)
All other	10 (1.7)

Subjects reported an average BB session duration of 19 minutes. They reported strong satisfaction with BB with all domains generating mean agreement ratings of 8.5 or higher, on a Likert scale of 0 (strongly disagree) to 10 (strongly agree), including: the ease of use of the website (8.4), good looking appearance (8.5), usefulness of information (9.2), and ease of learning the system (8.5). Twenty-four percent (24%) of subjects reported never using the website with a parent, 48% reported sometimes doing so, and 28% reported often or always using the website with a parent.

Results of the provider survey on experience with BB are shown in Table 5. Providers tended toward agreement that BB provided useful information; was easy to use; that their patients benefited from using BB; and that the asthma specialist feedback was useful. Responses were neutral on effectiveness of the discussion board for communication with patients, and on average, expressed neutral to slight disagreement on adequacy of time available to use BB, and if they had changed management based on BB data. Open-ended comments grouped into three consistent themes: the observation that BB improved communication with patients, the advantages of having access to information on patient asthma status on a more immediate, day to day, basis; and that the time commitment to use the system was a concern.

**Table 5:** **Post-project Provider Attitudes Towards BostonBreathes**

**Attitude statement**	Mean*, N = 14
BostonBreathes provided me with useful information about my patient(s) that I would not have had otherwise.	6.5
The BostonBreathes website was easy to use.	6.9
I had enough time in my schedule to use BostonBreathes.	4.6
I changed the asthma management of my patient(s) based on data from the site.	4.6
It was easy to remember to login to BostonBreathes.	5.0
I would recommend that other MDs use BostonBreathes for their asthma patients	5.6
BostonBreathes is best for severe asthmatics only.	5.0
I trusted the information being entered by my patient(s).	5.7
I believe my patient(s) benefited from using the BostonBreathes website.	6.6
I would recommend BostonBreathes for my asthmatic patients.	6.2
The discussion board was an effective way to communicate with my patient(s).	5.6
The feedback from Asthma Specialist posted to the discussion board was useful in helping me to manage my asthma patient(s).	6.3

*All answers rated on Likert scale 0-10 (0=Strongly Disagree to 10=Strongly Agree).

On average, subjects earned $6 (range, $0-$40) in incentive payments for website use over the 6 month participation period, plus $15.00 total in payments for completing pre and post study surveys.

## Discussion

BostonBreathes was designed to support multiple factors known to impact asthma morbidity including patient knowledge, medication adherence, and clinical teamwork. Key elements of feasibility [33] were successfully tested including: acceptability, and implementation. Overall adherence to preventer medications was not improved in either study group. Among the subset of subjects with low baseline compliance, the intervention demonstrated a significant improvement in adherence. Knowledge of the purpose of their own preventer medication improved significantly only among the small group of intervention subjects queried on this variable.

Although BB use tapered over time, the intensity of interaction with the website was adequate to suggest a possible favorable impact on both knowledge and adherence. Asthma symptoms and asthma-related behavioral variables decreased in both intervention and control groups. Although not all decreases were statistically significant, the patterns of decreases were similar for both groups for wheezing, night-time awakening, and parental loss of sleep. Additional exposure to the website and/or a longer follow-up period to allow benefits of improved adherence with controller medications to be manifested might be necessary to demonstrate down-stream favorable impact on symptoms. In a recent systematic review, all studies demonstrated that reminder systems increase adult patient medication compliance, but none improved measured clinical outcomes [34]. Other studies have shown that short term effectiveness of adult self-management guidance may not endure when assessed over long-term follow-up [35]. However, a recent adult study using web-based guided self-management interventions similar to this study demonstrated enduring benefits on several asthma outcomes [36]. It is possible that adults are more likely than children to persist in their utilization of web-based self-management tools, and thus more likely to show benefits in asthma morbidity measures.

The importance of teamwork among health care professionals caring for patients with chronic diseases is well recognized [17] and this study contributes to our understanding of how pediatric patients and their providers will utilize electronic communication technology. The majority of messages from health care providers were posted by the online asthma nurse, whereas the primary health care providers of the research subjects utilized the discussion boards at a low level, highlighting the challenges associated with engaging busy health care providers in case-management-related activities, and highlighting the essential role of an active case-manager. However, all team members, including patients, study nurse, asthma specialist, and PCPs, did demonstrate active use of the website communication functions, and the teamwork relationships established electronically, and supported by ready availability of relevant asthma symptom and medication use data, effectively facilitated periodic case review and feedback to the PCP by the asthma specialist. Feedback from providers emphasized that in the future the site might be used most efficiently by patients who most stand to benefit, such as those with persistent level symptoms and/or frequent use of the health care system. The site might also be managed by office staff responsible for case management of chronically ill patients, to reduce time demands on busy clinicians.

Although recent studies have focused on newer technologies such as smart-phone based monitoring and reminder systems [37], other recent research [1,16,36,38,39] continues to demonstrate the effectiveness, simplicity, and ease of use of web-based systems.

An important limitation of this study is its modest sample size resulting in limited statistical power. Several positive trends were identified but the number of subjects was not sufficient to definitively assess these areas. Other limitations include a consolidation of intervention groups which resulted in asymmetrically sized intervention and control groups, and erosion of utilization by intervention subjects. Control group subjects also demonstrated improvements in several domains, possibly due to an intervention effect from enrollment in the study and associated interactions with study staff and various measurements.

Delivering comprehensive asthma care to under-served populations is a challenging task in the setting of a brief, problem-focused primary care office visit. Implementation of accountable care organizations and medical home initiatives [40,41] and developing more effective models of caring for patients with chronic illnesses [42] will require new methods, like BB, for maintaining supportive clinical relationships outside of episodic in-person encounters between patients and their caregivers. Rapid changes in reimbursement models for health care services will continue to create demands for more effective support solutions for patients with chronic illnesses such as asthma.

## Conclusion

Findings from this study suggest that digital applications such as BostonBreathes have the potential to support multiple aspects of health care and health behavior change. Future work should use findings of this study to help identify the most engaging and effective design approaches, taking into consideration the developmental stages of pediatric users, while also prioritizing efficiency to insure systems are economically feasible on larger scales.

## References

[1] Chipps BE, Zeiger RS, Borish L, Wenzel SE, Yegin A, Hayden ML (2012). Key findings and clinical implications from the epidemiology and natural history of asthma: Outcomes and treatment regimens (TENOR) study. J Allergy Clin Immunol.

[2] Liu AH, Gilsenan AW, Stanford RH, Lincourt W, Ziemiecki R, Ortega H (2010). Status of asthma control in pediatric primary care: Results from the pediatric asthma control characteristics and prevalence survey study (ACCESS). J Pediatr.

[3] Bloomberg GR, Banister C, Sterkel R, Epstein J, Bruns J, Swerczek L (2009). Socioeconomic, family, and pediatric practice factors that affect level of asthma control. Pediatrics.

[4] Flores G, Snowden-Bridon C, Torres S, Perez R, Walter T, Brotanek J (2009). Urban minority children with asthma: Substantial morbidity, compromised quality and access to specialists, and the importance of poverty and specialty care. J Asthma.

[5] Clark NM, Dodge JA, Shah S, Thomas LJ, Andridge RR, Awad D (2010). A current picture of asthma diagnosis, severity, and control in a low-income minority preteen population. J Asthma.

[6] Bruzzese JM, Stepney C, Fiorino EK, Bornstein L, Wang J, Petkova E (2012). Asthma self-management is sub-optimal in urban hispanic and african American/black early adolescents with uncontrolled persistent asthma. J Asthma.

[7] Barnes PJ. Inhaled glucocorticoids for asthma. N Engl J Med. 1995;332(13):868–75. 10.1056/NEJM199503303321307 7870143

[8] Rank MA, Liesinger JT, Ziegenfuss JY, Branda ME, Lim KG, Yawn BP (2012). The impact of asthma medication guidelines on asthma controller use and on asthma exacerbation rates comparing 1997–1998 and 2004–2005. Ann Allergy Asthma Immunol.

[9] Desai M, Oppenheimer JJ (2011). Medication adherence in the asthmatic child and adolescent. Curr Allergy Asthma Rep.

[10] Krishna S, Francisco BD, Balas EA, Konig P, Graff GR, Madsen RW (2003). Internet-enabled interactive multimedia asthma education program: a randomized trial. Pediatrics.

[11] Wise M, Gustafson DH, Sorkness CA, Molfenter T, Staresinic A, Meis T (2007). Internet telehealth for pediatric asthma case management: Integrating computerized and case manager features for tailoring a web-based asthma education program. Health Promot Pract.

[12] Guendelman S, Meade K, Benson M, Chen YQ, Samuels S (2002). Improving asthma outcomes and self-management behaviors of inner-city children: a randomized trial of the health buddy interactive device and an asthma diary. Arch Pediatr Adolesc Med.

[13] Morrison D, Wyke S, Agur K, Cameron EJ, Docking RI, Mackenzie AM (2014). Digital asthma self-management interventions: a systematic review. J Med Internet Res.

[14] Huckvale K, Car M, Morrison C, Car J (2012). Apps for asthma self-management: a systematic assessment of content and tools. BMC Med.

[15] Christakis DA, Garrison MM, Lozano P, Meischke H, Zhou C, Zimmerman FJ (2012). Improving parental adherence with asthma treatment guidelines: a randomized controlled trial of an interactive website. Acad Pediatr.

[16] Meischke H, Lozano P, Zhou C, Garrison MM, Christakis D (2011). Engagement in “my child's asthma”, an interactive web-based pediatric asthma management intervention. Int J Med Inform.

[17] Wiecha J, Pollard T (2004). The interdisciplinary eHealth team: chronic care for the future. J Med Internet Res.

[18] Osman LM, Abdalla MI, Russell IT, Fiddes J, Friend JA, Legge JS (1996). Integrated care for asthma: matching care to the patient. Eur Respir J.

[19] The patient-centered medical home: history, seven core features, evidence, and transformational change. [Internet]; 2007. Available from: http://www.graham-center.org/online/etc./medialib/graham/documents/publications/mongraphs-books/2007/rgcmo-medical-home.Par.0001.File.tmp/rgcmo-medical-home.pdf.

[20] Institute of Medicine (2001). Crossing the quality chasm: a new health system for the 21st century.

[21] Zhao J, Zhai YK, Zhu WJ, Sun DX. Effectiveness of telemedicine for controlling asthma symptoms: A systematic review and meta-analysis. Telemed J E Health. 2014 Nov 13. [Epub ahead of print]. 10.1089/tmj.2014.0119 25393915

[22] Middleton AD (1997). Managing asthma: it takes teamwork. Am J Nurs.

[23] Harver A, Kotses H (2010). Asthma, health and society: a public health perspective.

[24] The ehealth behavior management model: a stage-based approach to behavior change and management. Prev Chronic Dis [serial online]. 2004; 1(4); 1-13. Available from: http://www.cdc.gov/pcd/issues/2004/oct/pdf/04_0070.pdf. PMC1277954 15670446

[25] The research-based web design & usability guidelines, Enlarged/Expanded edition. Washington: U.S. government printing office, 2006 [Internet]. Available from: http://www.usability.gov.

[26] Conn KM, Halterman JS, Fisher SG, Yoos HL, Chin NP, Szilagyi PG (2005). Parental beliefs about medications and medication adherence among urban children with asthma. Ambul Pediatr.

[27] Conn KM, Halterman JS, Lynch K, Cabana MD (2007). The impact of parents' medication beliefs on asthma management. Pediatrics.

[28] Bender B, Wamboldt FS, O'Connor SL, Rand C, Szefler S, Milgrom H (2000). Measurement of children's asthma medication adherence by self report, mother report, canister weight, and doser CT. Ann Allergy Asthma Immunol.

[29] Doser- from MEDITRACK PRODUCTS [Internet]. Available from: http://doser.com/.

[30] Division of Children’s Health Promotion, Department of Family Medicine, Georgetown University School of Medicine (2005). You can control asthma: a book for kids.

[31] Morgan WJ, Crain EF, Gruchalla RS, O'Connor GT, Kattan M, Evans R (2004). Results of a home-based environmental intervention among urban children with asthma. N Engl J Med.

[32] Diggle P (1989). Testing for random dropouts in repeated measurement data. Biometrics.

[33] Bowen DJ, Kreuter M, Spring B, Cofta-Woerpel L, Linnan L, Weiner D (2009). How we design feasibility studies. Am J Prev Med.

[34] Tran N, Coffman JM, Sumino K, Cabana MD (2014). Patient reminder systems and asthma medication adherence: a systematic review. J Asthma.

[35] Kauppinen RS, Vilkka V, Hedman J, Sintonen H (2011). Ten-year follow-up of early intensive self-management guidance in newly diagnosed patients with asthma. J Asthma.

[36] van Gaalen JL, Beerthuizen T, van der Meer V, van Reisen P, Redelijkheid GW, Snoeck-Stroband JB (2013). Long-term outcomes of internet-based self-management support in adults with asthma: randomized controlled trial. J Med Internet Res.

[37] Vasbinder EC, Janssens HM, Rutten-van Molken MP, van Dijk L, de Winter BC, de Groot RC (2013). E-monitoring of asthma therapy to improve compliance in children using a real-time medication monitoring system (RTMM): the e-MATIC study protocol. BMC Med Inform Decis Mak.

[38] Araujo L, Jacinto T, Moreira A, Castel-Branco MG, Delgado L, Costa-Pereira A (2012). Clinical efficacy of web-based versus standard asthma self-management. J Investig Allergol Clin Immunol.

[39] van der Meer V, Bakker MJ, van den Hout WB, Rabe KF, Sterk PJ, Kievit J (2009). Internet-based self-management plus education compared with usual care in asthma: a randomized trial. Ann Intern Med.

[40] Bitton A, Martin C, Landon BE (2010). A nationwide survey of patient centered medical home demonstration projects. J Gen Intern Med.

[41] Nutting PA, Crabtree BF, Miller WL, Stewart EE, Stange KC, Jaen CR (2010). Journey to the patient-centered medical home: a qualitative analysis of the experiences of practices in the national demonstration project. Ann Fam Med.

[42] Coleman K, Austin BT, Brach C, Wagner EH (2009). Evidence on the chronic care model in the new millennium. Health Aff (Millwood).

